# Prioritization of control measures in leakage scenario using Hendershot theory and FBWM-TOPSIS

**DOI:** 10.1371/journal.pone.0298948

**Published:** 2024-04-05

**Authors:** Fereydoon Laal, Amirhossein Khoshakhlagh, Saber Moradi Hanifi, Mostafa Pouyakian

**Affiliations:** 1 Social Determinants of Health Research Center, Department of Occupational Health Engineering, Birjand University of Medical Sciences, Birjand, Iran; 2 Department of Occupational Health Engineering, Faculty of Health, Kashan University of Medical Sciences, Kashan, Iran; 3 Department of Occupational Health Engineering, School of Public Health, Iran University of Medical Sciences, Tehran, Iran; 4 Department of Occupational Health and Safety Engineering, School of Public Health and Safety, Shahid Beheshti University of Medical Sciences, Tehran, Iran; Istanbul University: Istanbul Universitesi, TURKEY

## Abstract

Currently, there is increasing concern about the safety and leakage of process industries. Therefore, the present study aims to prioritize control measures before and after the leakage scenario by using the Hendershot theory and MCDM techniques. In this study, two proactive and reactive layers were selected before and after leakage of tanks, respectively. Then, criteria and alternatives were selected to perform fuzzy TOPSIS (FTOPSIS) and find the best alternative based on the literature review and Hendershot approach. The linear model of the fuzzy Best-Worst method (FBWM) was constructed and resolved using Lingo 17 software. Subsequently, criteria were assigned weights based on thorough calculations of the inconsistency rate. The weight of study experts was equal to 0.25. The results of FBWM showed that the reliability index with a weight of 0.3727 was ranked first and the inconsistency rate ($\tilde{\xi }*/\xi$) was calculated to be equal to 0.040. Inherent Safety Design (ISD) (0.899) and passive safety (0.767) also ranked first before and after tank leaks, respectively. Using the FBWM method leads to fewer pairwise comparisons and at the same time more stability. Although ISD and passive strategies are more valid and strict, elements of all strategies are necessary for a comprehensive process safety management program.

## 1. Introduction

Process facilities such as oil companies, refineries, and petrochemical industries store a large amount of flammable and dangerous chemicals in storage tanks [1]. The leakage of these substances has been one of the threatening factors for workers and residents around these industries and the environment [2]. Therefore, it seems more necessary to pay attention to preventive and control measures.

### 1.1 History and function of safety barriers

From the very dawn of human existence, mankind has relied on safety barriers as a means to safeguard both individuals and their precious possessions against the relentless threats posed by adversaries and natural hazards. During the period of industrialization, the emergence of man-made hazards necessitated the implementation of safety barriers to avert any accidents that may arise from these dangers. The notion of safety barriers often finds its correlation within an accident model known as the energy model [3]. It is indispensable to recognize that Gibson (1961) took precedence in pioneering the advancement of this energy model [4], while Haddon’s significant contribution lies in his development and consideration of ten strategic approaches toward preventing accidents by this model [5].

In recent times, there has been a significant shift in the understanding of safety barriers. Hollnagel highlights the modern concept of defense in depth, which encompasses various forms of protective measures (ranging from preventing the release of radioactive substances to incident reporting and safety protocols) [6]. While initially developed within the nuclear industry, this idea has now found application in other high-risk industries like process industries where multiple layers of protection are also employed [3]. Additionally, recognized standards including IEC:61508 (1998), IEC:61511 (2002), and ISO:13702 (1999) emphasize that safety barriers play a crucial role in mitigating accident risks [3].

ISO 13702 presents a set of definitions intended to elucidate the concepts of prevention, control, and mitigation of safety. Prevention is defined as the act of diminishing the likelihood of a hazardous event occurring. Control refers to exerting measures that curtail either the extent or duration of such an event, thus preventing it from escalating further. Mitigation, on the other hand, involves efforts aimed at alleviating the consequences incurred by a hazardous event [3, 7]. Both prevention and control strive to minimize both the probability and scope of any given hazardous occurrence. In essence, these actions seek to preemptively hinder such events from transpiring or rapidly quell their progression if they do arise. Meanwhile, mitigation endeavors pertain solely to reducing adverse impacts emanating from said dangerous happenings. The root causes behind unwanted incidents often stem from a combination of technical malfunctions, human errors, and external occurrences that harbor potential risks. Accidents are events that often occur unintentionally and unexpectedly and cause human, financial, and environmental damages [3, 8].

Hollnagel explicates the primary functions of safety barriers as encompassing prevention and protection. These barriers are implemented before an anticipated event, serving as a preventive measure. It is assumed that these barriers prevent the accident or slow down its progress on the way to becoming an accident. The barriers considered after the occurrence of a specific event are used as a means of protection and protect the environment, people, and systems from the consequences of the accident [6].

Svenson (1991) classifies barriers into various categories including physical systems, technical aspects, and human-organizational factors [9], while Neogy et al. divide them into physical, procedural, or administrative classifications [10]. Reason (2016) argues that administrative controls constitute a significant component of defense systems and can be categorized into two main types: external controls which consist of laws, regulations, and procedures outlining proper actions and methodologies to be followed; internal controls glean knowledge and principles through training and experience [11]. Risk mitigation criteria in IEC: 61511 are also categorized as follows: safety instrument systems (SIS), systems related to technological safety, and external risk mitigation facilities [3].

One of the risk control patterns is Hendershot’s hierarchy of control strategies, which is often used in process industries [12–14]. Hendershot classified strategies into four distinct groups for mitigating the frequency or consequences of potential incidents: Inherent, Passive, Active, and Procedural. The risk control approaches found within the first two categories, inherent and passive, are deemed more reliable and robust due to their reliance on the physical and chemical properties of the system itself rather than being contingent upon the successful operation of tools, devices, and procedures [12, 13].

### 1.2 Literature review (Hendershot theory and prioritization with MCDM)

Researchers in process safety analysis mainly face three types of uncertainty: completeness, modeling, and parameter [15]. In these studies, they have tried to use different methods to reduce these uncertainties [16–18]. Hajiaghaei et al. used the Pythagorean Fuzzy Technique for Order Preference by Similarity to the Ideal Solution (PF-TOPSIS) method to select the best Green Supplier Selection (GSS) [19]. Erdebilli et al. also used Q-Rung Orthopair Fuzzy (Q-ROF) Fuzzy TOPSIS, and VIKOR methods to select sustainable private health insurance policies [20]. Gul et al used a hybrid approach based on a Bayesian Best-Worst method (BWM) and Fuzzy VIKOR in an oil station [21]. Lin et al. also performed a safety evaluation of the drilling system through MCDM modeling based on TOPSIS in the fuzzy environment [22].

In this study, a hybrid approach was presented for prioritizing proactive and reactive control measures based on the Hendershot theory. Therefore, Fuzzy, TOPSIS, and FBWM methods were used to reduce uncertainty and choose the best ideal solution with the least complexity due to their strengths which are described below. Fuzzy theory is a suitable tool for ambiguous and uncertain conditions that can convert qualitative expressions into numerical probabilities [22]. Multi-criteria decision-making (MCDM) methods were developed in various studies and have solved complex problems in management sciences [16]. AL-baker et al used MCDM techniques such as entropy and weighted sum methods under the authority of the Single Value Neutrosophic (SVN) Scale [23]. Mohamed and Ismail proposed the use of α-D MCDM to address various supply chain evaluation problems [24]. One of the important topics is management and decision-making issues in the field of safety [25]. Different approaches have been used for prioritization and weighting in the studies. In the decision domain, a popular approach to acquiring the final ranking solution is based on distance. TOPSIS, VIKOR, CODAS, etc. are among the techniques that use the distance-based approach for rank evaluation [16]. Therefore, Fuzzy TOPSIS (FTOPSIS) was used in this study as well.

In the TOPSIS method, the opinions of the decision-makers are expressed in the form of definite numbers, while the decision-makers cannot determine the exact weight of the criteria and score the alternatives according to each criterion, and it is always accompanied by some errors and ambiguity. The TOPSIS technique is used to rank and compare various alternatives, choose the best option, and determine the distance between the options [26]. This method offers several advantages such as accommodating criteria or indicators with different units of measurement as well as those exhibiting negative or positive qualities. Within this technique lies the concept that an ideal solution will provide maximum benefits while incurring minimal costs [26, 27].

The BWM, a novel addition to the realm of Multiple Criteria Decision Making (MCDM), was introduced by Rezaei in 2015. This approach derives its essence from pairwise comparisons, wherein the emphasis lies on determining the priority level of the best criteria over other factors, while also favoring all criteria collectively in contrast to the worst attribute with a scale ranging from 1 to 9 [28]. The BWM method curtails the need for excessive comparative data and engenders more robust evaluations, thereby proffering more dependable outcomes [28, 29]. Accordingly, this study utilizes this methodology to ascertain the weightage attributed to TOPSIS criteria.

### 1.3 Study objectives

Therefore, this study proposes a comprehensive integrated approach to evaluate risk control solutions using the Hendershot theory, FTOPSIS, and BWM. In this approach, the number of pairwise comparisons is reduced and Hendershot’s quadruple approaches are prioritized. Thus, this hybrid approach provides a lower computational requirement with higher accuracy. Also, combining fuzzy logic with TOPSIS provides more realistic results with less uncertainty. The results of this research can be used as a guide for making scientific decisions and choosing appropriate strategies to control the consequences of leakage in floating roof tanks.

## 2. Material and methods

**Fig 1:**
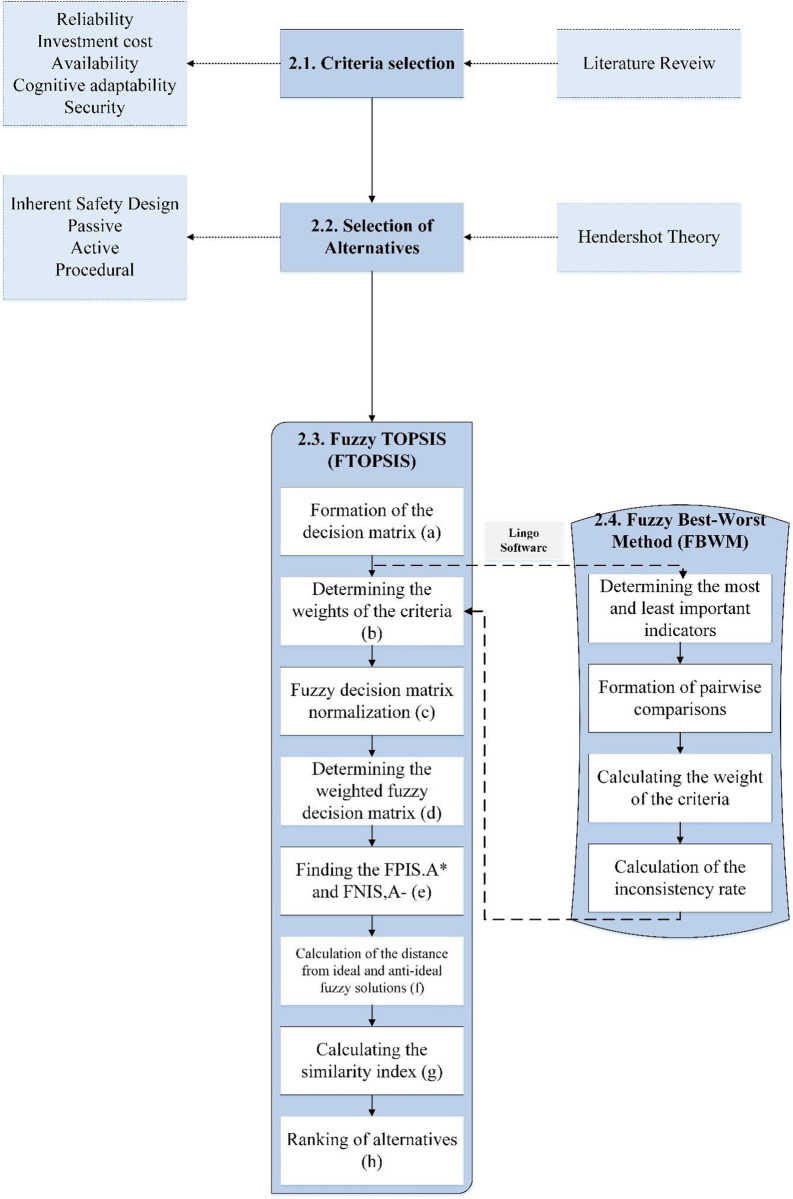
Study flowchart.

The current study is descriptive-analytical research that was conducted on the tank leakage scenario of a petrochemical company in Iran. Fig 1 shows the current study, which is discussed below.

In this study, FTOPSIS was used to prioritize protective layers and controls. Two proactive and reactive layers were selected before and after methanol leakage, respectively. First, criteria and alternatives were selected to perform FTOPSIS and find the best alternative. Therefore, 5 final criteria were selected by reviewing the literature. Then, 4 main alternatives were presented as process safety strategies according to the basic events of the study and based on the Hendershot approach [14]. The weighting of the criteria was also done using the fuzzy BWM (FBWM) method. Then, the FTOPSIS and FBWM questionnaires and pairwise comparisons were given to the experts after selecting the criteria and alternatives. Four study experts were selected from the same industry. Meanwhile, verbal consent was obtained from the experts. Then, the selection of the ideal solution was done in two situations before and after the main scenario (methanol leak).

### 2.1 Criteria selection

At this stage, various related studies were examined to select the criteria, which were reduced to 5 criteria according to the opinions of the research team and experts, because it is necessary to conduct the FTOPSIS study and provide independent criteria (Table 1).

**Table 1:** FTOPSIS criteria.

Row	Criteria	Description
1	Reliability	To measure the option’s ability to perform the relevant tasks accurately, when required [30–32]
2	Investment cost	Estimated cost for implementing the options [31, 33]
3	Availability	The option’s ability to perform well when run for a targeted period before or beyond the update to reduce operational downtime [33]
4	Cognitive adaptability	Dynamic, flexible, and modifiable option according to changes [34]
5	Security	Protection of system infrastructure from vandalism and terrorism [35, 36]

### 2.2 Selection of alternatives

In this study, the Hendershot method [13, 14] was used to select the alternatives. According to Hendershot’s opinion, process safety strategies are divided into four categories based on efficiency according to Table 2.

**Table 2:** The strategies (alternatives) studied before and after the occurrence of the main scenario [13, 14].

Alternatives	Alternatives (strategy)	Description	Examples in the present study
A1	Inherent Safety Design (ISD)	ISD was introduced as the most effective strategy in the 70s by Keltz who states that risks can be identified before the implementation phase of process plans, and early elimination of risks, improves the safety level of the system [37–39]. Keltz proposed the basics of the inherent safety of chemical processes in the four principles of Intensification, substitution, Attenuation, and simplification [37, 40].	Absence of High High (HH) Alarm on PT, The sound frequency of the alarm is the same
A2	Passive	Arrangements in which the features of equipment and process design are well considered from the beginning can reduce the risk of possible accidents without interfering with the active operation of any other equipment. The appropriate location for the construction of the project is one of the passive safety issues.	Inadequate performance of firewalls, process conditions, and weather
A3	Active	In active safety, process safety control equipment and systems such as sensors and detectors, instrumentation equipment, artificial intelligence, and automatic fire alarm and extinguishing systems (sprinklers) are used to actively control system safety. These systems are designed to detect a dangerous situation and perform an appropriate reaction.	Breathing valve failure, sprinkler failure, solenoid valve failure
A4	Procedural	It includes a set of safe work instructions, safety rules, operator training, emergency response instructions, and management systems.	Failure to issue PTW, defects in the restart, incorrect job description, inadequate audit and standard operating procedures (SOP)

### 2.3 Fuzzy TOPSIS

During this phase, the panel of experts selected criteria that fit the objectives. The alternatives were also divided into 4 categories: Inherent Safety Design (ISD), passive, active, and procedural based on the Hendershot theory. Then, the steps of FTOPSIS were performed to select the best alternatives. In addition, FBWM was used to weight the criteria. The FTOPSIS method was presented by Chen and Hwang, which was created by converting the TOPSIS presented by Hwang and Yoon into a fuzzy state [41]. This stage has several basic steps from (a) to (h) according to Fig 1. Its steps are as follows:

### a. Formation of the decision matrix

The decision matrix is fashioned through Eq 1 by taking into account the number of criteria, alternatives, and assessment of each alternative across various criteria.


$$\tilde{D}=\left[\begin{array}{cccc} \tilde{x_{11}} & \tilde{x_{12}} & \cdots & \tilde{x_{1n}}\\ \tilde{x_{21}} & \tilde{x_{22}} & \ldots & \vdots \\ \vdots & \vdots & \cdots & \vdots \\ \tilde{x_{m1}} & \tilde{x_{m2}} & \ldots & \tilde{x_{mn}} \end{array}\right]$$ (1)

In this study, triangular fuzzy numbers were used for simplicity in decision-making. In this case $\tilde{x_{ij}}=(a_{ij},b_{ij},c_{ij})$, is the performance of alternative i (i = 1,2,…,m) to criterion j (j = 1,2,…,n). If the team of decision makers has k members, and the fuzzy ranking of the kth decision-maker $\tilde{x_{ijk}}=(a_{ijk},b_{ijk},c_{ijk})$, for i = 1,2,…,m and j = 1,2,…,n, the combined fuzzy ranking $\tilde{x_{ij}}=(a_{ij},b_{ij},c_{ij})$ of the alternatives can be acquired by Eqs 2 to 4.


$$a_{ij}=\begin{array}{r} \mathrm{Min}\\ k \end{array}\left(a_{ijk}\right)$$ (2)

$$b_{ij}=\frac{\sum_{k=1}^{k}b_{ijk}}{k}$$ (3)

$$c_{ij}={Max}_{k}^{\left(c_{ijk}\right)}$$ (4)

### b. Determining the weights of the criteria

In this phase, the crucial weighting factor associated with various criteria in the process of decision-making is delineated by Eq 5.


$$\tilde{w}=(\tilde{w_{1}},\tilde{w_{2}},\ldots ,\tilde{w_{n}})$$ (5)

If the team of decision makers has k members, and the importance coefficient of the kth decision-making member, $\tilde{w_{jk}}=(w_{jk1},w_{jk2},w_{jk3})$, for j = 1,2,…,n, the combined fuzzy ranking $\tilde{w_{j}}=(w_{j1},w_{j2},w_{j3})$ can be acquired by Eqs 6 to 8.


$$w_{j1}=\begin{array}{r} \mathrm{Min}\\ k \end{array}\left(w_{jk1}\right)$$ (6)

$$w_{j2}=\frac{\sum_{k=1}^{k}w_{jk2}}{k}$$ (7)

$$w_{j3}={Max}_{k}^{\left(c_{jk3}\right)}$$ (8)

### 2.4 Fuzzy Best-Worst Method (FBWM)

In this study, FBWM was used before step (b) to determine the weight of the criteria using Lingo 17 software, and due to its importance, we considered it as a separate step. According to the findings by Guo and Zhao (2017), it was determined that when decision-makers in BWM engage in qualitative judgments, particularly through pairwise comparisons on a 1–9 scale, inherent ambiguity tends to prevail. In light of this observation, they proposed FBWM as a means to effectively capture and represent the perplexing nature of human judgments with regard to uncertainty and ambiguity [42]. It is summarized in 4 steps.

#### Step 1: Determining the most and least important indicators

In the initial phase of the FBWM, it is crucial to identify both the most significant (best) and least significant (worst) indicators. In this particular study, these indicators were determined initially by seeking the opinions of research experts.

#### Step 2: Formation of pairwise comparisons

During this stage, a series of pairwise comparisons are conducted between the finest criteria and all other criteria (Best to others (BO)), as well as between all other criteria and the weakest criteria (Others to Worst (OW)). In this particular investigation, experts were provided with pairwise comparisons and asked to ascertain their degree of preference using a 5-point fuzzy spectrum. This spectrum comprises verbal expressions ranging from equally important (EI), weakly important (WI), fairly important (FI), very important (VI), to absolutely important (AI). Table 3 shows the concepts of weighting and membership function.

**Table 3:** Concepts of weighting and membership function [42].

Linguistic term	Triangular fuzzy numbers (TFNs)
EI	1,1,1
WI	2.3,1,3.2
FI	3.2,2,5.2
VI	5.2,3,7.2
AI	7.2,4,9.2

#### Step 3: Calculating the weight of the criteria

In this subsequent stage, the intricate nonlinear optimization model of the problem was duly formulated using the following relationship. However, according to the study of Rezaei and Guo and Zhao, models with three or more criteria should be converted into linear models [28, 42]. Thus, the linear model of the FBWM method was established and diligently resolved using the Lingo 17 software. Subsequently, the weights associated with each criterion were successfully acquired.


$$\begin{array}{l} {min\;\tilde{\xi }}^{*}\\ s.t.\;\left\{ \begin{array}{c} \left|\frac{\left(l_{B}^{w},m_{B}^{w},u_{B}^{w}\right)}{\left(l_{j}^{w},m_{j}^{w},u_{j}^{w}\right)}-(l_{Bj},m_{Bj},u_{Bj})|\leq (k^{+},k^{+},k^{+}\right)\\ \left|\frac{\left(l_{j}^{w},m_{j}^{w},u_{j}^{w}\right)}{\left(l_{W}^{w},m_{W}^{w},u_{W}^{w}\right)}-(l_{jW},m_{jW},u_{jW})|\leq (k^{+},k^{+},k^{+}\right)\\ \sum_{j=1}^{n}R(\tilde{w_{j}})=1\\ l_{j}^{w}\leq m_{j}^{w}\leq u_{j}^{w}\\ l_{j}^{w}\geq 0\\ j=1,2,\ldots ,n \end{array}\right. \end{array}$$ (9)

The fuzzy weight was acquired through the direct solution of the aforementioned model using the Lingo software. Subsequently, these vague weights were transformed into determined weights utilizing Eq 10.


$$R({\tilde{a}}_{i})=\frac{l_{i}+{4m}_{i}+u_{i}}{6}$$ (10)

#### Step 4: Calculation of the inconsistency rate

In this step, the inconsistency rate of the pairwise comparisons of the research was calculated. First, we calculated the unknown value ξ using Eq 11, by solving a quadratic equation. Based on this the consistency index was obtained. Subsequently, we divided the optimally determined value of the objective function ($\tilde{\xi }*$) relating to every linear model utilized for these paired comparison tables by said compatibility index to procure what is referred to as an incompatibility rate. Expressing this mathematically: ($(\tilde{\xi }*)/\xi$) equates to determining said inconsistency rate. The nearer the rate of inconsistency approaches zero, the greater the consistency of the pairwise comparison.


$$\xi^{2}-(1+{2u}_{BW})\xi +(u_{BW}^{2}-u_{BW})=0$$ (11)

### c. Fuzzy decision matrix normalization

The calculation of the dimensions of the normalization of the Fuzzy decision matrix for both positive and negative criteria is contingent upon Eqs 12 through 15. These equations methodically employ triangular fuzzy numbers.

Positive side:

$$\tilde{r_{ij}}=\left(\frac{a_{ij}}{{c*}_{j}},\frac{b_{ij}}{{c*}_{j}},\frac{c_{ij}}{{c*}_{j}}\right)$$ (12)

Negative side:

$$\tilde{r_{ij}}=\left(\frac{{\bar{a}}_{j}}{c_{ij}},\frac{{\bar{a}}_{j}}{b_{ij}},\frac{{\bar{a}}_{j}}{a_{ij}}\right)$$ (13)

Where,

$${c*}_{j}={Max}_{i}^{\left(c_{ij}\right)}$$ (14)

$${\bar{a}}_{j}={Min}_{i}^{\left(a_{ij}\right)}$$ (15)

### d. Determining the weighted fuzzy decision matrix

The weighted fuzzy decision matrix shall be obtained through the multiplication of the significance coefficient associated with each criterion in the unscaled fuzzy matrix, as expressed by Eq 16.


$$\tilde{V_{ij}}={\tilde{r}}_{ij}.{\tilde{w}}_{ij}$$ (16)

Where ${\tilde{w}}_{j}$ expresses the importance coefficient of the C_j_ criterion. Thus, the matrix will be in the form of Eq 17.


$$\tilde{V}={\left[{\tilde{v}}_{ij}\right]}_{m\times n}$$ (17)

For positive and negative criteria, we act as follows (Eqs 18 and 19).


$${\tilde{v}}_{ij}=\frac{a_{ij}}{c_{j}^{*}}.w_{j1},\frac{b_{ij}}{c_{j}^{*}}.w_{j2},\frac{c_{ij}}{c_{j}^{*}}.w_{j3}$$ (18)

$${\tilde{V}}_{ij}=\frac{{\bar{a}}_{j}}{c_{ij}}.w_{j1},\frac{{\bar{a}}_{j}}{b_{ij}}.w_{j2},\frac{{\bar{a}}_{j}}{a_{ij}}.w_{j3}$$ (19)

### e. Finding the Fuzzy Positive Ideal Solution (FPIS.A*) and the Fuzzy Negative Ideal Solution (FNIS,A^-^)

The FPIS.A* and the **FNIS,A**^**-**^ are defined as Eqs 20 and 21, respectively.


$$A^{*}=({\tilde{V}}_{1}^{*},{\tilde{V}}_{2}^{*},{\ldots ,V}_{n}^{*})$$ (20)

$$A^{-}=({\tilde{V}}_{1}^{-},{\tilde{V}}_{2}^{-},{\ldots ,\tilde{V}}_{n}^{-})$$ (21)

That ${\tilde{V}}_{i}^{*}$ is the best value of criterion i among all alternatives and ${\tilde{V}}_{i}^{-}$ is the worst value of criterion i among all alternatives. These values are obtained from Eqs 22 and 23.


$$v_{j}^{*}={Max}_{i}^{\left({\tilde{v}}_{ij3}\right)}$$ (22)

$${\tilde{v}}_{j}^{-}={Min}_{i}^{\left({\tilde{v}}_{ij1}\right)}$$ (23)

The alternatives that are placed in A* and A- indicate completely better and completely worse alternatives, respectively.

### f. Calculation of the distance from ideal and anti-ideal fuzzy solutions

These distances can be calculated from Eqs 24 and 25, respectively.


$$S_{i}^{*}=\sum_{j=1}^{n}d({\tilde{v}}_{ij},v_{j}^{*})$$ (24)

d (…) is the distance between two fuzzy numbers that if (a1, b1, c1) and (a2, b2, c2) are two triangular fuzzy numbers, this distance is obtained from Eq 25.


$$D_{v}({\tilde{M}}_{1},{\tilde{M}}_{2})=\sqrt{\frac{1}{3}({\left(a_{1}-a_{2}\right)}^{2}+{\left(b_{1}-b_{2}\right)}^{2}+{\left(c_{1}-c_{2}\right)}^{2})}$$ (25)

### g. Calculating the similarity index (Eq 26)

$${CC}_{i}=\frac{S_{i}^{-}}{\left(S_{i}^{*}+S_{i}^{-}\right)}$$ (26)

### h. Ranking of alternatives

In this stage, the alternatives are ranked according to the similarity index, so that the alternatives with the most similarity index are given priority. Meanwhile this investigation constitutes a segment of a larger research endeavor, which has been deemed ethically sound under the code IR.SBMU.PHNS.REC.1398.003 by Shahid Beheshti University of Medical Sciences.

## 3. Results

### 3.1 Results of FTOPSIS

In this study, the FBWM and FTOPSIS methods were used to achieve the best control strategy. First, the weights of the criteria were calculated using the FBWM, and then the alternatives before and after the main scenario (methanol leak) were ranked using the FTOPSIS method. Table 4 shows the demographic features of the experts in the paired comparison questionnaire in terms of gender, age, work experience, and education. All expert groups had almost a weight of 0.25. Therefore, at this stage, the weight factor will not affect the results.

**Table 4:** The weighting factor of experts in FTOPSIS.

Variables	Experts’ scores (Score)			
	Expert 1	Expert 2	Expert 3	Expert 4
**Organization titles**	Occupational Health and Safety (3)	Senior expert in analyzer and instrumentation (3)	fire chief (4)	Master of Process Safety (3)
**Work experience(years)**	9 (2)	13 (3)	16 (3)	17 (3)
**Education level**	PhD (5)	Master of Science (4)	Bachelor of Science (3)	Master of Science (4)
**Age(years)**	36 (2)	36 (2)	39 (2)	39 (2)
**Expert weight score**	12	12	12	12
**Weight factor (WF)**	0.250	0.250	0.250	0.250

### 3.2 Determining the weights of the study criteria using the FBWM

The studied indicators are reliability (C1), investment cost (C2), availability (C3), cognitive compatibility (C4), and security (C5).

#### Step 1: Determining the most important and least important indicators

In the first step of the best-worst method, using the opinions of research experts, the most important (best) and least important (worst) indicators were identified, which respectively included reliability (C1) and investment cost (C2).

#### Step 2: Formation of pairwise comparisons

In this particular section, pairwise comparisons of the BO and OW were performed. Following this exploration, a paired comparison questionnaire was distributed among 10 esteemed experts to ascertain their preference levels based on a comprehensive spectrum outlined on a five-point fuzzy table. Pairwise comparisons, after responding, were pooled by the geometric mean method, as described below (Table 5).

**Table 5:** Pairwise comparisons of the best and worst criteria.

Criteria	C1	C2	C3	C4	C5
**Best (C1)**	-	(3.059, 3.565, 4.070)	(1.936, 2.449, 2.958)	(1.273, 1.741, 2.257)	(1.976, 2.479, 3.006)
**Worst (C2)**	(3.059, 3.565, 4.070)	-	(0.844, 1.231, 1.748)	(0.917, 1.320, 1.840)	(1.234, 1.692, 2.218)

#### Step 3: Calculating the weight of the criteria

At this stage, the linear optimization model of the problem had taken shape within the FBWM (Fuzzy Best-Worst Method), subsequently resolved using the Lingo software version 17. Consequently, the weights corresponding to each criterion were obtained and duly presented in Table 6.

**Table 6:** Weight and final rank of the indicators.

Criteria	Fuzzy weight	Definite weight	Rank
**Reliability (C1)**	(0.288, 0.406, 0.324)	0.3727	1
**Investment cost (C2)**	(0.086, 0.124, 0.104)	0.1143	5
**Availability (C3)**	(0.099, 0.188, 0.129)	0.1633	4
**Cognitive consistency (C4)**	(0.127, 0.199, 0.184)	0.1845	2
**Security (C5)**	(0.119, 0.173, 0.172)	0.1638	3

The fuzzy weights were derived through the direct solution of the model within Lingo software (as detailed in Table 6). Then, these fuzzy weights were transformed into definitive forms. For instance, the fuzzy weight of the reliability index and its definite weight are equal to (0.324, 0.406, 0.288) and 0.3727, respectively. Based on this, the reliability index with a weight of 0.3727 has been ranked first. Also, the indices of cognitive compatibility (C4) and security (C5) were ranked second and third with weights of 0.1845 and 0.1638, respectively.

#### Step 4: Calculation of the incompatibility rate

At this point, the inconsistency rate of the pairwise comparisons was computed by the prescribed method. The findings revealed that ξ, which denotes the unknown value of the consistency index, amounted to 7.47. Subsequently, we divided the optimal value of the objective function ($\tilde{\xi }*$) of each linear model for pairwise comparison tables by this aforementioned compatibility index to derive the incompatibility rate ($(\tilde{\xi }*)/\xi$). In our present analysis, this value yielded 0.040. It is worth noting that a smaller inconsistency rate indicates a greater degree of compatibility within the particular pairwise comparison being assessed [28, 42]. The compatibility rate of pairwise comparisons also shows the stability of experts’ opinions.

### 3.3 The results of the FTOPSIS method before and after the methanol leakage scenario

The ranking of the research alternatives before and after the methanol leakage was done according to Table 2, the results of which are given below. The distance of the alternatives from the ideals was done after forming the decision matrix, normalizing it, weighting the normal matrix, and determining positive and negative ideals. Then the distance of each alternative from the positive ideal (D^+^) and the negative ideal (D^-^) was calculated. The results can be seen in Tables 7 and 8, the second and third columns. The similarity index and ranking of alternatives were also done according to Tables 7 and 8. The higher the similarity index of an alternative, the better the ranking of that alternative.

**Table 7:** Final ranking of alternatives (before methanol leakage).

Alternative	The distance to the positive ideal	The distance to the negative ideal	Final score	Rank
**A1**	0.0301	0.269	0.899	**1**
**A2**	0.0815	0.235	0.742	**3**
**A3**	0.0655	0.234	0.782	**2**
**A4**	0.2966	0.00	0.0001	**4**

**Table 8:** Final ranking of alternatives (after methanol leakage).

Alternative	The distance to the positive ideal	The distance to the negative ideal	Final score	Rank
**A1**	0.0301	0.269	0.899	**1**
**A2**	0.0815	0.235	0.742	**3**
**A3**	0.0655	0.234	0.782	**2**
**A4**	0.2966	0.00	0.0001	**4**

According to Table 7, the ISD strategy ranked first among the 4 strategies before the methanol spill. Active and passive safety strategies were ranked second and third respectively.

According to Table 8, the passive safety strategy is ranked first after the methanol leakage. Inherent safety and active strategies were ranked second and third respectively. In addition, a 50% increase in weights was applied to analyze the sensitivity of the results, and the ranking did not change.

## 4. Discussion

In this study, FTOPSIS was used to determine the best control strategy. The FBWM method was used for weighting the criteria. Also, because the expert group weighted 0.25, its effect was considered the same. The most and least important indicators, using the opinions of experts, respectively, included the reliability criteria (C1) and investment cost (C2). Finally, the criteria of reliability (C1), cognitive compatibility (C4), security (C5), availability (C3) and cost (C2) were ranked one to five respectively.

According to the results, the use of the FBWM method leads to fewer pairwise comparisons and more stable comparisons, or in other words, provides more reliable results [28]. The number of pairwise comparisons in AHP, is equal to (n(n-1)/2) [33], while these comparisons are reduced to (2n-3) in FBWM [28]. Thus, only reference comparison should be done in this method. The basis of these comparisons is to evaluate the relative fuzzy preference of the best criteria over other criteria and all criteria over the worst criteria. Karimi et al. (2020) reported the satisfactory performance and high efficiency of this method and concluded that this method works better than fuzzy AHP [43].

According to the results of the FTOPSIS method, ISD and passive safety strategies were the most important alternatives before and after the methanol leakage, respectively. The results showed that to create a preventive and appropriate approach, the most important principle is ISD. Applying the principles of inherent safety in the early phases of process design is very efficient because it is cheap and efficient. Therefore, the most important decisions regarding the ISD principles should be made in the initial design phases [44]. As the development and design of the process progress, the chance of applying the principles of inherent safety decreases. Therefore, the first step for this type of design is to identify all the risks in the process. The main idea of ISD is to improve safety by eliminating or reducing (internal strategy) rather than controlling or managing (external strategy) risks [38]. Accidents related to chemical storage can have a domino effect, so one should not just focus on the ISD feature of the system. As the results of the present study also showed, risks should be reduced through the next three layers of protection (active, passive, and procedures) [45].

Formulation of laws, guidelines, and standards is one of the common methods for managing risk and improving the safety of work environments [46]. Unfortunately, less attention has been paid to the issue of ISD in Iran. Jafari et al.’s study (2018) further demonstrated that the adoption of ISD philosophy in Iran is marred by numerous impediments [47]. Failing to acknowledge and address these hurdles can detrimentally impact the successful acceptance of ISD. Within this research, a comprehensive blueprint was ingeniously devised, employing index-based methodologies, to effectively gauge the inherent safety of the process at hand [47].

According to the study of Reniers and Amyotte, ISD is at the top of the hierarchy of process safety strategies. ISD is considered one of the main prevention paths in the future to prevent losses in chemical and process industries to reduce or eliminate risks in the early stages of design [48].

The next most important strategy or control layer was active safety. In active safety, equipment and process safety control systems such as sensors and detectors, instrumentation equipment, artificial intelligence, etc. are used to actively control system safety. These systems are designed to detect a dangerous situation and perform an appropriate reaction. In this study, most of the process errors such as breathing valve failure, sprinkler failure, solenoid valve failure, etc. are related to this department. Therefore, the need to pay special attention to this part will play an effective role in preventing risks in the processes after the ISD. As the technology space expands and increases in complexity, it requires more than procedural, passive, and active risk mitigation strategies to achieve optimal risk reduction due to potential deficiencies using guidelines, and physical safety barriers [49].

After the main scenario, passive safety was identified as the most important control strategy, according to experts’ opinions. Therefore, measures should be taken to reduce the probability and severity of possible accidents without relying on the active operation of any other equipment. The location chosen for project construction is an important factor to consider in ensuring passive safety. If the tanks are properly situated, the likelihood of domino accidents will be minimized.

An important finding of this study was the potential for the domino effect caused by the fire of the tanks to spread to nearby petrochemicals. Reniers and Khakzad also demonstrated that paying attention to risks both inside and outside the site, and arranging the tanks in the best possible way, can significantly reduce the domino effects of accidents [50]. Reniers and Khakzad proposed a method that utilizes BN, LUP, and AHP to evaluate the location of new facilities and modifications in existing facilities, based on the associated risk level [50]. However, it is essential to recognize that incorporating ISD and passive approaches adds further validity and rigor to the study. All four approaches are indispensable for the development of a comprehensive safety management program.

### 4.1 Uncertainty

Various methods have been employed to reduce uncertainty in different studies. In this particular study, we sought to reduce uncertainty by utilizing fuzzy logic, BWM, TOPSIS, and expert weighting. Fuzzy logic enables the reduction of uncertainty in expressing probabilities, by employing multi-valued logic instead of two-valued logic [20]. It is considered a suitable approach to safety and risk management, as it addresses both unpredictability and qualitative variables [51]. Fuzzy logic utilizes expert insights to assess probabilities associated with key events that have been highlighted in numerous studies [52]. The combination of fuzzy logic with different approaches provides more realistic results with less uncertainty [53].

There are several applications of fuzzy set theory to reduce uncertainty and inaccuracy in expert judgment, including triangular, intuitive, trapezoidal, and Gaussian fuzzy numbers [54, 55]. The choice of membership function depends on the nature of the problem [56]. Therefore, triangular fuzzy numbers were used in this study because they can easily solve the problem under some weak assumptions [57]. In various studies, triangular and trapezoidal fuzzy numbers have been used due to their flexibility and simplicity [57].

The decision-making process requires information. The important thing about this information is its validity and reliability [58]. In the real world, most of the information has uncertainty and this uncertainty should be taken into account in the decision-making process [59]. Fuzzy theories alone cannot fully include this uncertainty in calculations [59, 60]. The level of confidence varies according to different experts, and the uncertainty and difference in the validity of their opinions cannot be ignored [61]. The advantage of Z-Numbers compared to conventional fuzzy methods is to consider uncertainty in the opinion of experts and assign credit to their opinion for estimating fuzzy parameters [62, 63]. This issue was one of the limitations of the present study, which is recommended to be considered in future studies.

### 4.2 Managerial insights

The results of the present study can be used in the process of decision-making and prioritization of control measures of storage tanks. Safety systems by operational procedures use management controls [64]. These systems include operating procedure standards, safety rules, operator training, emergency response procedures, and management systems. However, executive procedures alone cannot control risk in high-risk systems. Because the reliability of the human factor is not very high, and people usually cannot diagnose the problem and take the necessary actions quickly enough [14]. Executive procedures are always a part of a comprehensive management system due to their ability to check preventive and periodic maintenance and manage systems based on engineering control solutions. ISD also has high reliability [14, 65]. It removes the risk and is not considered a protective layer. Therefore, it can be used as the best control solution.

## 5. Conclusion

In the present study, the FTOPSIS approach, Hendershot theory, and FBWM were used to prioritize and weight the study criteria, respectively. The best control strategies were selected with a preventive and protective approach before and after the main scenario based on the Hendershot theory. The results showed that the use of the approach of the present study reduced the number of pairwise comparisons and the results were presented with higher reliability because the reference comparison of the best and worst criteria was evaluated. In addition, the use of fuzzy logic and its combination with TOPSIS and BWM provides more realistic results with less uncertainty. Also, study experts were weighted due to their different different insights into the problem. The most important alternatives with preventive and reactive approaches included ISD and passive safety, respectively. Reliability (C1) and investment cost (C2) were assigned the most weight using the FBWM. ISD was chosen as the most important layer based on the results of FTOPSIS, but due to the occurrence of domino events in storage tanks, other control layers (active, passive, and procedures) in the Hendershot theory should also be considered. Therefore, despite the validity of ISD and passive strategies, elements of all strategies are necessary to implement a comprehensive safety management program. The results of this research can be used as a guide for making scientific decisions and choosing the right strategy for prioritizing control criteria in process industries, especially storage tanks. The uncertainty in experts’ opinions was one of the limitations of the present study, which is recommended to be corrected in future studies by using other approaches such as Z-numbers.
